# Identification of m6A modification patterns and development of m6A–hypoxia prognostic signature to characterize tumor microenvironment in triple-negative breast cancer

**DOI:** 10.3389/fimmu.2022.978092

**Published:** 2022-08-29

**Authors:** Xi Shen, Jianxin Zhong, Jinlan He, Jiaqi Han, Nianyong Chen

**Affiliations:** ^1^ Department of Head and Neck Oncology and Department of Radiation Oncology, Cancer Center, West China Hospital, Sichuan University, Chengdu, China; ^2^ Department of Breast Oncology, Key Laboratory of Carcinogenesis and Translational Research (Ministry of Education), Peking University Cancer Hospital & Institute, Beijing, China

**Keywords:** Triple-negative breast cancer, m6A RNA methylation, m6A-hypoxia signature, tumor microenvironment, immune cell infiltration

## Abstract

**Background:**

N6-methylation (m6A) modification of RNA has been found to have essential effects on aspects of the tumor microenvironment (TME) including hypoxia status and mobilization of immune cells. However, there are no studies to explore the combined effect of m6A modification and hypoxia on molecular heterogeneity and TME of triple-negative breast cancer (TNBC).

**Methods:**

We collected The Cancer Genome Atlas (TCGA-TNBC, *N*=139), the Molecular Taxonomy of Breast Cancer International Consortium (METABRIC-TNBC, N=297), the GSE103091, GSE21653, and GSE135565 series from the Gene Expression Omnibus (GEO-TNBC, N=247), and FUSCCTNBC (N=245) for our study. The non-negative matrix factorization algorithm was used to cluster TNBC samples. Immune cell infiltration was analyzed by the CIBERSORT algorithm. The enrichment scores were calculated by single-sample gene set enrichment analysis(ssGSEA) to characterize TME in TNBC samples. Immunohistochemistry (IHC) and qRT-PCR were performed to detect the gene expression.

**Results:**

Based on the expression of m6A-related genes, we identified three distinct m6A clusters (denoted A, B, and C) in TNBC samples. Comparing the TME characteristics among the three clusters, we observed that cluster C was strongly related to hypoxia status and immune suppression, whereas clusters A and B displayed more immune cell infiltration. Therefore, we combine m6A and hypoxia related genes to classify two m6A-hypoxia clusters of TNBC and screened six prognostic genes by LASSO-Cox regression to construct a m6A-hypoxia signature(MHPS), which divided TNBC samples into high- and low-risk groups. We identified different TME features, immune cell infiltration between the two groups, and a better immunotherapy response was observed in the low-risk group. A nomogram was constructed with tumor size, lymph node, and risk score to improve clinical application of MHPS.

**Conclusion:**

We identified distinct TME characteristics of TNBC based on three different m6A modification patterns. Then, we constructed a specific m6A–hypoxia signature for TNBC to evaluate risk and predict immunotherapy response of patients, to enable more accurate treatment in the future.

## Introduction

Breast cancer is the most common female malignant cancer, with an estimated global annual mortality of 41,760 in women, and represents a highly heterogeneous disease (1). Triple-negative breast cancer (TNBC), a subtype that lacks estrogen receptor, progesterone receptor, and HER2 expression, accounts for 10-20% of all breast cancers. TNBC exhibits the most malignant biological behaviors, including high levels of proliferation and a high degree of immune infiltration, and has the worst prognosis (2, 3). In contrast to ER+/HER2+ breast cancers, where targeted therapies or endocrine therapies may be used, the treatment of TNBC still relies on surgery and chemotherapy. Therefore, a more comprehensive exploration of the molecular mechanisms of TNBC is required to enable development of more effective therapies (4).

N6-methylation of adenosine (m6A) is the most common epigenetic modification in mammals. This modification occurs within the typical consensus sequence RRACH (where R = G or A, and H = A, C, or U) and is controlled by m6A methyltransferases (“writers”) and m6A demethylases (“erasers”); there are also binding proteins that decode m6A methylation, termed “readers” (5, 6). Such m6A RNA modifications have been identified in diverse cancers including breast cancer, lung cancer, and leukemia (7–9). Compelling evidence indicates that m6A modification can reversibly modulate RNA splicing and stability, as well as translation of crucial oncogenes, leading to tumor development. Therefore, we aimed to identify the effects of m6A modification patterns on the biology of TNBC to facilitate the development of more precise therapeutic approaches.

The tumor microenvironment (TME), which consists of tumor cells, inflammatory cells, and extracellular matrix components, is essential for tumor development (10). Research has also shown that m6A regulators have an influence on the TME. For instance, in gastric tumor, the characteristics of immune cells infiltrating the TME were found to depend in the expression of 21 m6A regulators (11). Wang et al. (12) showed that METTL3-mediated m6A modification could promote the activation and maturation of dendritic cells (DCs), and that depletion of YTHDF1 could strengthen the ability of DCs to present tumor antigens and enhance the infiltration of CD8+ T cells in tumors (13). Immunotherapy is currently an emerging treatment for TNBC, and its effects have not yet been clearly demonstrated in patients. In previous studies, we showed that the expression of three immune checkpoints, cytotoxic T lymphocyte-associated protein 4 (CTLA-4), programmed cell death protein 1 (PD-1), and programmed death-ligand 1 (PD-L1) which was regulated by m6A modification. For example, METTL3/IGF2BP3 axis mediated m6A modification of PD-L1 mRNA and mobilized infiltrating immune cells to resist tumor progression (14, 15).

Hypoxia of the TME causes an insufficient supply of oxygen and nutrients to tumor cells, which leads to arrest of tumor cell apoptosis, as well as promote proliferation and secretion of pro-angiogenic growth factors (16). In prostate and cervical cancers, tumor hypoxia has been identified as an independent indicator of poor prognosis and a factor in resistance to radiotherapy and chemotherapy (17, 18). Moreover, many studies have reported that m6A genes participate in the formation of a hypoxic microenvironment. For example, FTO, an m6A regulator, promoted the progression of hypoxic TME formation *via* effects on glucose metabolism through FOXO1 mRNA expression (19). In turn, tumor hypoxia regulated the function of m6A reader YTHDF1 to drive the malignancy of hepatocellular carcinoma (20). Hypoxia of the TME is a characteristic of a “cold” immune state in almost all solid cancers and is associated with unsatisfactory results of immune checkpoint inhibitor (ICI) therapy. Therefore, given the important and closely related effects of m6A modification and TME hypoxia, we aimed to identify m6A-hypoxia genes and characterize immune infiltration, to enable development of a targeting strategy that could compliment ICI therapy.

Many studies have been devoted to elucidating the molecular mechanisms of specific m6A regulators. Here, we describe the relationship between m6A genes and characteristics of the TME in samples from TNBC patients collected from The Cancer Genome Atlas (TCGA), the Gene Expression Omnibus (GEO), and METABRIC. We use the non-negative matrix factorization (NMF) algorithm and single-sample gene set enrichment analysis (ssGSEA) to characterize the relationships of TME hypoxia with m6A clusters and develop an m6A-hypoxia gene signature to predict overall survival and immunotherapy response.

## Materials and methods

### Data analysis of TNBC samples

All data were downloaded from freely accessible public databases. We obtained both gene somatic mutation information and copy number variation data from TCGA and METABRIC dataset *via* the CBioPortal website (https://www.cbioportal.org/). For gene expression, we obtained both the TCGA-TNBC gene transcription profile and corresponding clinical information using the “TCGAbiolinks” R package. We standardized the raw data to FPKM (fragments per kilobase of transcript per million mapped reads) by calculating gene lengths and the total numbers of reads mapped to protein-coding genes (20). Three GEO data microarrays, GSE103091, GSE21653, and GSE135565, with clinical information were acquired by “GEOquery” R package. Based on the GLP570 sequencing platform, we removed the differences in batch effects between datasets, integrated GSE103091, GSE21653, and GSE135565 into a new cohort through the ComBat function from the “sva” R package and selected the samples with negative immunohistochemistry for ER, PR, and HER2 as GEO-TNBC (21). RNA-seq of FUSCCTNBC((OEZ000398)) was downloaded from NGDC platform.

For this study, we collected 139 TCGA-TNBC cases, 297 BRCA-METABRIC cases, 245 FUSCCTNBC cases, and 274 GEO-TNBC cases with an overall survival longer than >30 days for further analysis. Transcriptome sequencing for two human TNBC cell lines (MDA-MB-231 and SUM159) under normoxia and hypoxia was obtained from GSE144569.

### Cluster analysis of 46 m6A-related genes

These 46 m6A-related genes consisted of 21 m6A regulators (writers: METTL3, METL14, METL16, WTAP, KIAA1429, ZC3H13, RBM15, and RBM15B; readers: YTHDC1, YTHDC2, YTHDF1, YTHDF2, YTHDF3, HNRNPC, HNRNPA2B1, IGFBP1, IGFBP2, IGFBP3, and RBMX; and erasers: FTO and ALKBH5) and 25 related genes which were differently expressed (|Fold Change|>2, p.value<0.05) between TNBC and normal tissues and screened by Pearson’s correlation analysis (|cor. r|>0.4, p.value<0.05) (ANLN, BCL2, CDKN2A, CEBPB, COL4A5, DGAT2, EGFR, FASN, FOSB, FOXM1, FSCN1, HSPB1, IGF1R, IGF2, IGFBP5, LGR6, MYB, PPARA, RAR, RYR1, SALL2, SERPINE2, SFRPQ, SRRK1, and THSD4), and used to classify TNBC samples into subtypes by an unsupervised NMF algorithm as previously described (22). The NMF analysis aimed to identify the gene expression profiles of samples and cluster the gene expression matrix into different groups through the “CancerSubtypes” R package (22). This package was used to calculate the silhouette width to evaluate the clustering stability, and for overall survival analysis to predict prognosis when the samples were divided into three clusters (23).

### TME estimation, biomarkers analysis, and functional annotation

The ssGSEA algorithm was used to analyse 255 tumor-related signatures, including metabolism, TME, and tumor development, with the “IOBR” R package (23). Signatures related to the TME(Nature_metabolism_hypoxia, Hu_hypoxia, TMEscoreA_plus), immune (immune_checkpoints, antigen processing and presenting machinery (APM)), DNA_replication, DNA damage_repair(DDR), and tumor cell biology(epithelial-mesenchymal transition (EMT), pan-fibroblast TGFb response, Ferroptosis, and exosomal_secretion) were used to characterize the biological features of TNBC clusters (24, 25). To estimate the proportions of infiltrating immune cells in TNBC samples, we used the CIBERSORT algorithm in R, which enables to identify 22 immune cell types in samples including regulatory T cells (Tregs), gamma delta T cells, follicular helper T cells, CD8 T cells, naive CD4 T cells, resting CD4 memory T cells, activated CD4 memory T cells, plasma cells, resting natural killer (NK) cells, and activated NK cells (25). We also extracted and visualized the expression of immune checkpoint biomarkers (CD86, TNFRSF9, IDO1, ICOS, CTLA4, PD1, and PDL-1) and hypoxia biomarkers (CA9, FOSL1, HILPDA, MRGBP, SLC2A1, and VEGFA) in TNBC clusters using the “ggpubr” R package (26). We assess the immune, stromal, ESTIMATE scores and tumor purity of TNBC patients with the ESTIMATE algorithm.

To explore the differences in biological processes between the three clusters, the gene set “h.all.v7.0.symbols” was downloaded from MSigDB, and enrichment scores were calculated using the “GSVA” R package (27). Gene ontology (GO) analysis was performed using Metascape (https://metascape.org/gp/index.html).

### Identification of differentially expressed genes to determine m6A modification patterns and hypoxia status

Based on the differences in m6A modifications and hypoxia status among TNBC samples, we screened out the m6A-DEGs among the three different m6A-based clusters and obtained their intersection with the hypoxia-DEGs between hypoxic and normoxia samples with the same criterion |Fold Change|>0.5, p.value<0.05 using the “limma” R package. Finally, 26 genes were selected as m6A-hypoxia genes and used to characterize two TNBC gene clusters using the “CancerSubtypes” R package (22).

### Construction of m6A-hypoxia gene signature (MHPS)

The m6A-hypoxia genes obtained as described above were then subjected to univariate Cox regression analysis to gain 21 m6A-hypoxia genes associated with TNBC overall survival, with p.value<0.05 for further analysis. Then, LASSO-Cox analysis was performed to identify the final six m6A-hypoxia genes for TNBC prognostic signature based on the smallest partial likelihood deviance. Finally, a signature of 6 m6A-hypoxia genes was established to calculate the risk score of TNBC patients, and the formula was shown below:


$$\text{risk score}=\sum_{i=1}^{n}Coef_{I}Expression_{i}$$

where n, Coef_i_, and Expression_i_ represent the number of signature genes, the coefficient index, and the gene expression level, respectively.

The “survival” and “glmnet” R packages were used to performed LASSO-Cox regression analysis of the m6A-hypoxia genes. The “survminer” R package was used to classify high- and low-score groups using the optimal cut-off point according to the maximally selected log-rank statistics. A receiver operating characteristic (ROC) curve was also generated for assessment of the predictive ability of the signature using the “pROC” R package. To evaluated the clinical effects of signature scores in TNBC, we included risk scores and other clinicopathological characteristics and constructed a nomogram to predict 1-, 3-, and 5-year overall survival using the “rms” R package.

### Estimation of immunotherapeutic response between MHPS groups

ICI immunophenoscore (IPS) values for the TCGA-TNBC samples were obtained from The Cancer Immunome Atlas Database. As IPS is considered to be a good index for measuring tumor immunogenicity, we estimated the correlations between IPS and m6A-hypoxia gene signature groups in order to evaluate the immunotherapeutic effects of immune checkpoint inhibitors(ICI). The expressions levels of CTL4A, PD-1, and PD-L1 in MHPS groups were compared. In addition, a immune checkpoint blockade treatment cohort (IMvigor210 for PD-1 treatment) was obtained, and the corresponding normalized data were utilized to analyze whether MHPS could estimate the ICIs response.

### Cell culture and quantitative real-time PCR

The MDA-MB-231(from Rolf Brekken at UT Southwestern Medical Center, Dallas, TX, in 2015) and SUM159(from Gregg L. Semenza at Johns Hopkins School of Medicine, Baltimore, MD, in 2014) cells were exposed to 20% or 1% O2 for 24 hrs from GSE144569. In our study, we obtain the cells from iCell Bioscience Inc, Shanghai, China and cultured the normal mammary epithelial cells MCF10A and triple negative breast cancer cells MDA-MB-231 with specific medium(Pricella, CM-0525) and L15(biosharp, BL313A) in a humidified atmosphere of 5% CO2 and 20% O2 at 37°C. The primer pairs used in qRT‐PCR were as follows: PIM2 5’-CCTGATAGACCTACGCCGTG-3’(Forward primer) and 5’-TGCATGGTACTGGTGTCGAG-3’(Reverse primer),PET117 5’-CTAGGAGCTCGAAGGTGGTG-3’(Forward primer) and 5’-GTCACGAAGCCTCTGCTGG-3’(Reverse primer), ABCB10 5’-GTCCCTATCGCCGTTCACTG-3’(Reverse primer) and 5’-GACAGTCAGAGTGTTCCGCT-3’(Forward primer), TAF9 5’-CCGCGGTTAAGTGTTGGTTC-3’(Reverse primer) and 5’-GGGACATGGGAGTCCCTACT-3’(Forward primer), MKP1 5’-TAGCAATCCCGTTGCCAAGT-3’(Reverse primer) and 5’-ATTCTCCACAGTGTCTGCCG-3’(Forward primer). Total RNA was extracted by Trizol (Invitrogen, USA). The PrimeScript^®^ RT Master Mix Perfect Real Time kit (TAKARA) was used to synthesize DNA. PCR amplifications were performed with the SYBR PremixEx Taq II (TAKARA).

### Immunohistochemistry to detect protein expression in tissue samples

We collected twenty pairs of TNBC tissues and adjacent normal breast tissues from Peking University Cancer Hospital. The study was conducted in accordance with the Declaration of Helsinki. All samples were collected with the written informed consent of the patients, and the study was approved by the Ethics Committee of Peking University Cancer Hospital. The tissues were fixed with 10% formalin, embedded by paraffin, and sectioned. Then we selected the optimal tissue sections for degreasing and immunohistochemistry staining. Antibodies used in this study are as follows: PIM2(abcam, ab107102), MKP-1(Novus Biologicals, NBP2-67909).

### Statistical analysis

Survival analysis was performed using Kaplan-Meier curves, which were performed and visualized using the “ggsurvplot” R package. The “maftools” R package was used to display gene mutation profiles of TNBC samples (28). The differences of clinical features among different clusters were tested by Pearson’s chi-square analysis, and Wilcoxon test or Kruskal-Wallis test to determine statistically significant differences between two or multiple clusters. Student’s t-tests was used to identify the different expression level of hub genes between normal breast cell line and TNBC cell line in qRT-PCR assay. p<0.05 was considered to indicate statistical significance in this study. (*p< 0.05; **p< 0.01: ***p< 0.001: ****p< 0.0001).

## Results

### Association between m6A modification and TME signatures in TNBC

**Figure 1:**
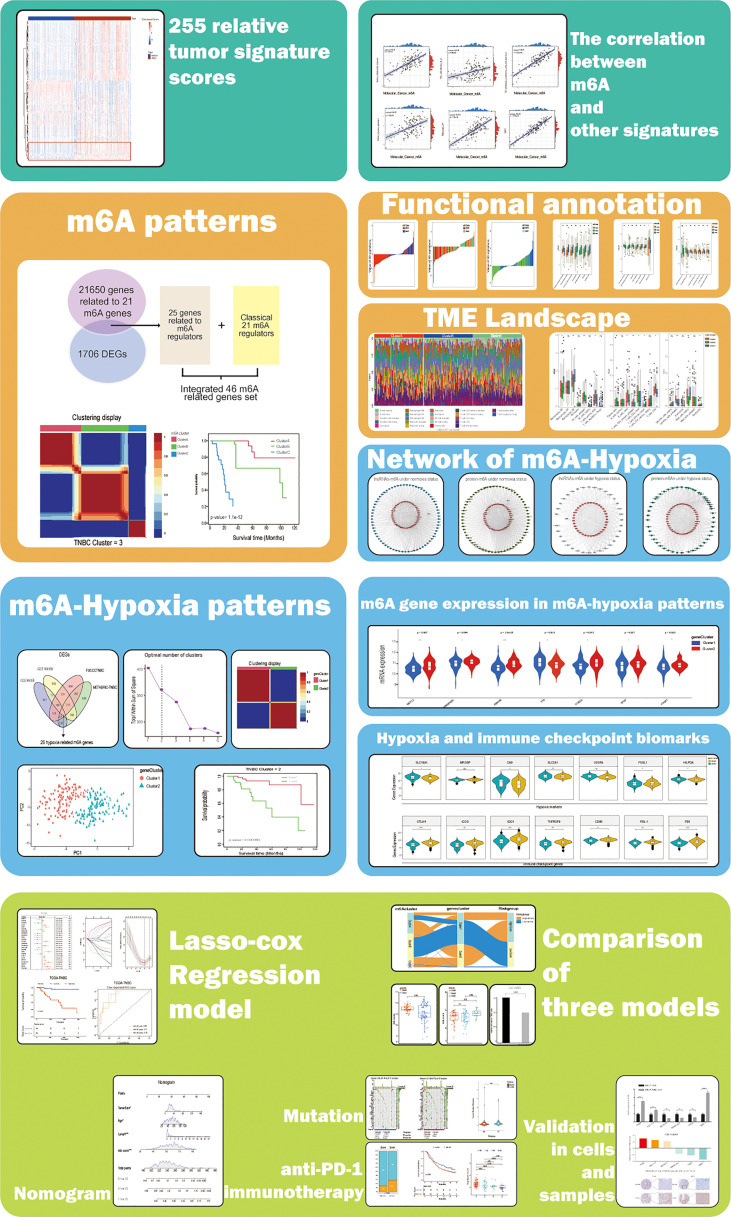
Study flow chart.

**Figure 2:**
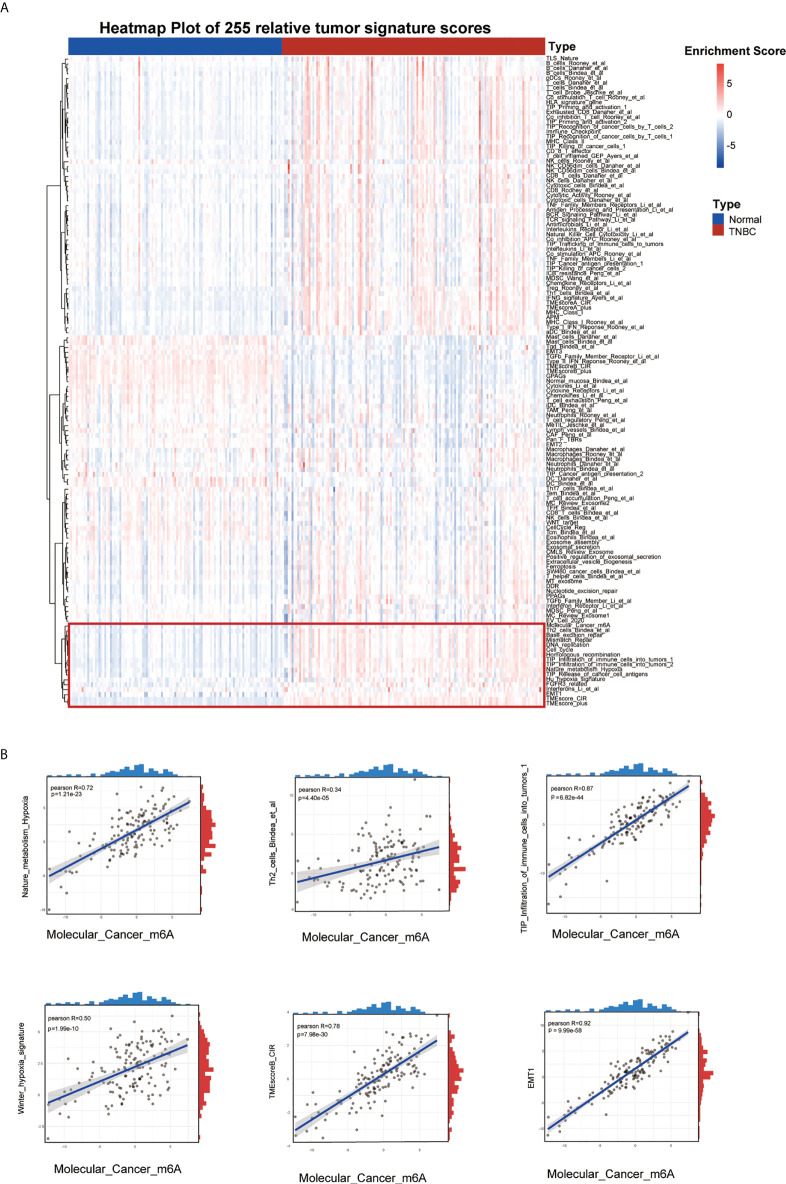
Landscape of tumor relative signatures in triple-negative breast cancer. **(A)** The heatmap of 255 tumor relative signatures score of 139 TNBC cases and 114 normal cases from TCGA. **(B)** The positive correlation between Molecular_Cancer_m6A and Nature_metabolism_Hypoxia, Winter_hypoxia_signature, Th2_cells_Bindea_et_al, TMEscoreB_CIR, Tip_Infiltration_of_immune_cells_into_tumors_1 and EMT.

The flow chart of our study is shown in **Figure 1**. Firstly, we scored 255 signatures including TME-associated, tumor-metabolism, and tumor-intrinsic signatures in 139 TCGA-TNBC samples and 114 normal tissue to explore TNBC biological features (**Figure 2A**). Through correlation analysis, we found that the Molecular_Cancer_m6A signature (11) was significantly associated with the following signatures: TIP_Infiltration_of_immune_cells_into_tumors_1 (29), Th2_cells_Bindea_et_al (30), EMT1 (31), Nature_metabolism_Hypoxia (32), Winter_hypoxia_signature (33), and TMEscoreB_CIR (34). These results indicated that m6A modification was strongly associated with the hypoxia and immune microenvironment in TNBC development (**Figure 2B**). Furthermore, the Molecular_Cancer_m6A signature, which consisted of eight m6A writers (METTL3, METL14, METL16, WTAP, KIAA1429, ZC3H13, RBM15, and RBM15B), eleven m6A readers (YTHDC1, YTHDC2, YTHDF1, YTHDF2, YTHDF3, HNRNPC, HNRNPA2B1, IGFBP1, IGFBP2, IGFBP3, and RBMX), and two m6A erasers (FTO and ALKBH5) showed differential expression between TNBC tissues and normal tissue (**Supplementary Figure 1A**). In addition, we analyzed the genetic alterations of 21 m6A regulators and found worse prognosis and higher risk of recurrence in patients with alterations in these genes; moreover, the samples with such alterations were more likely to be distributed in luminal B, HER2+, and TNBC (basal-like) subtypes of breast cancer(**Supplementary Figures 1B-D**). Of the 349 TNBC samples from METABRIC and TCGA with alterations in the m6A regulation genes, 31.4% of them had somatic mutations of m6A genes and 80.8% had copy number variants of m6A genes (**Supplementary Figure 1E, F**). These results suggested that there existed distinct m6A modification patterns such as m6A related signature network and genetic alteration of m6A regulators in TNBC patients.

### Classification of m6A modification clusters in TNBC patients

**Figure 3:**
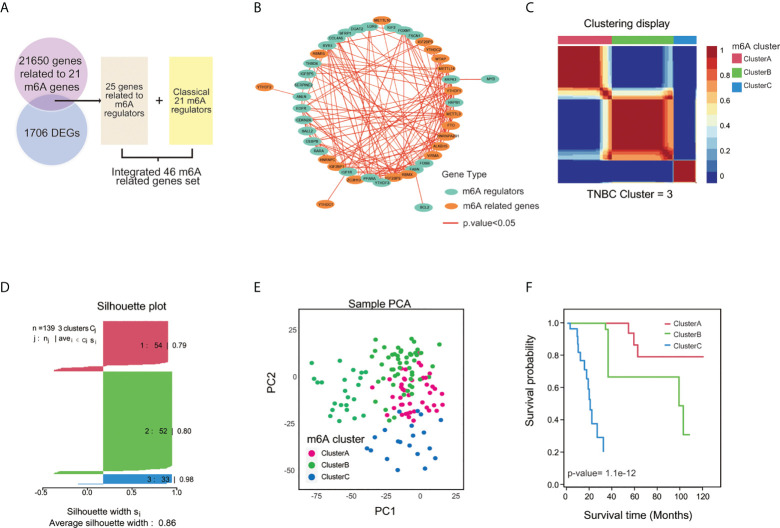
Clustering TNBC based on m6A-related genes by NMF analysis. **(A)** The flow diagram illustrated the process of selecting 46 m6A-related genes. **(B)** The correlation network of 46 m6A-related genes among 139 TNBC cases with p.value<0.05. **(C)** Three m6A clusters by Principal Component Analysis (PCA). **(D)** TNBC samples were clustered by the Nonnegative Matrix Factorization (NMF) method. **(E)** Silhouette width plots of NMF analysis. **(F)** K-M curve for comparing the overall survival among three clusters by the “CancerSubtypes” package.

The roles of the 21 m6A regulators have been investigated in several cancers. However, the m6A molecular modification is a dynamic reversible regulatory process that involves many genes. To fully explore m6A modification in TNBC, 21,650 genes related to m6A regulators were collected from the literature, and identified 25 genes specifically expressed in TNBC, with |Fold Change|>2 and p.value<0.05 compared with normal sample, were included for the following analysis (**Figure 3A**; **Supplementary Figure 2A**). The relationships between the 21 m6A genes and 25 related genes were estimated by Spearman analysis (**Figure 3B**; **Supplementary Figure 2B**). GO analysis also showed that the 46 m6A-related genes (21 m6A regulators and 25 m6A-related genes) contributed to RNA m6A methylation and transportation(**Supplementary Figure 2C**). The 46 m6A related genes were subjected to NMF analysis to characterize three m6A clusters (A,B and C) with gene expression data in all TNBC cohorts (TCGA-TNBC, GEO-TNBC, FUSCCTNBC, and METABRIC-TNBC) (**Figure 3C**; **Supplementary Figures 3A-C**). The silhouette width plots showed a value of 0.86 value for silhouette width, suggesting a better match between TNBC subtypes compared with other classifications of TNBC (**Figure 3D**). PCA also revealed that TNBC patients in A, B and C m6A subgroups were distinctively clustered (**Figure 3E**). Survival analysis showed that patients in m6A cluster C had worse overall survival than m6A clusters A and B according to K-M curves (p.value=1.1e-12) (**Figure 3F**). These results were validated in the GEO-TNBC, FUSCCTNBC, and METABRIC-TNBC cohorts (**Supplementary Figures 3D-F**). The significantly differential expression of 46 m6A related genes among the three TNBC m6A clusters (**Supplementary Figure 4**) suggests that m6A modification might have different roles in different clusters.

### Functional annotation revealing the association between m6A clusters and TNBC biological characteristics

**Figure 4:**
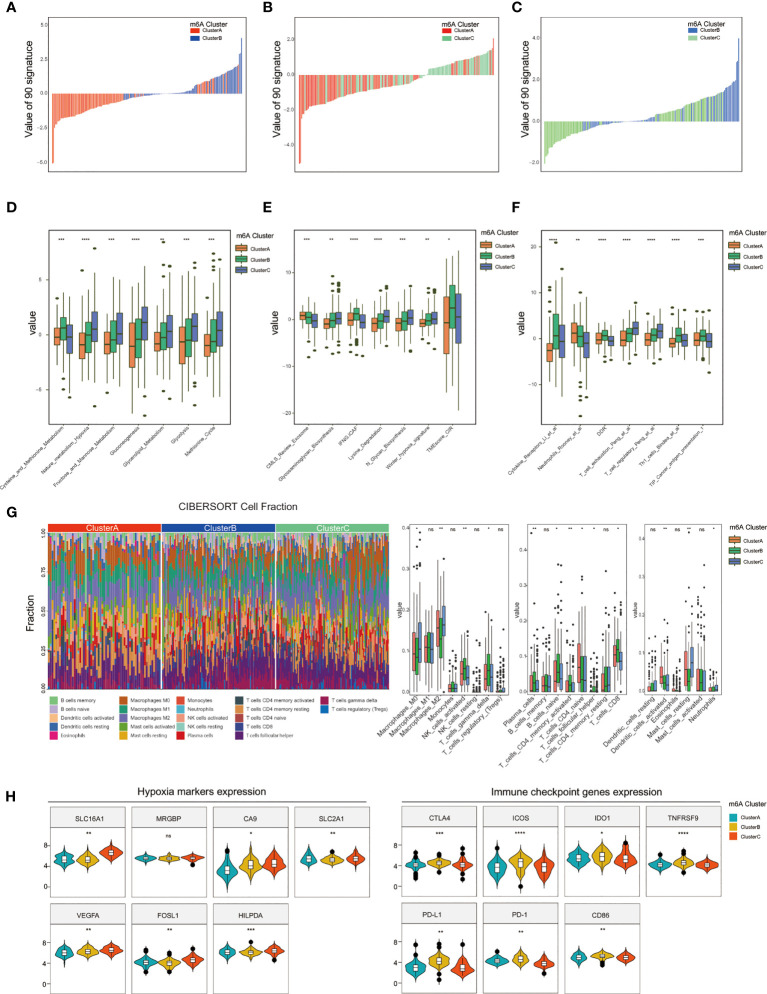
Comparison of TME and immune infiltration among m6A clusters. **(A–C)** The scores of 90 signatures among three m6A clusters. **(D–F)** Identification of 21 signatures differently scored in both m6A cluster1, m6A cluster2, and m6A cluster3. **(G)** Estimation and comparison of the immune infiltrated cells among three m6A clusters. **(H)** Distinct expression of hypoxia and immune checkpoint biomarkers between m6A cluster1, m6A cluster2, and m6A cluster3. (*P < 0.05,**P < 0.01, ***P < 0.001, and ****P < 0.0001). ns, not significant.

To depict the relationship between biological characteristics associated and different m6A subtypes, 255 signatures based on TME-associated, tumor-metabolism, and tumor-intrinsic signatures were used to score the three m6A clusters among 274 GEO-TNBC samples according to expression matrix. Then we screened 90 signatures with the criteria of |fold change|>=0.5, p.value<0.05 by intersecting the m6A modification related signatures among m6A clusters A, B, and C (**Supplementary Figure 5A**). The distributions of 90 intersection signatures scores between m6A cluster A vs m6A cluster B, m6A cluster A vs m6A cluster C, and m6A cluster B vs m6A cluster C were different, which suggested that different m6A modification patterns in the three clusters had distinctly influence on tumor characteristics(**Figures 4A–C**). By Kruskal-Wallis analysis, we identified 21 signatures with significantly different values(p<0.05) among the three m6A clusters. The signatures associated with tumor malignant phenotype such as Nature_metabolism_hypoxia, Glycolysis, methionine cycle, Winter_hypoxia_signature, T_cell_exhaustion and T_cell_regulation signature presented a higher enrichment for cluster C than cluster A and B(**Figures 4D–F**). The results indicated that m6A cluster C had stronger metabolism activities of glucose and amino acids, closer association with hypoxia microenvironment, and present a immune suppression phenotype. In addition, we used the CIBERSORT algorithm to estimate patterns of immune cell infiltration in the three m6A clusters. The results were coincident with above that activated NK cells, gamma delta T cells, plasma cells, naive B cells, activated CD4 memory T cells, and CD8 T cells were more likely to infiltrate in m6A cluster B, which also suggested that cluster A and B had a better anti-tumor immune response(**Figure 4G**). We further explored the gene expression of biomarkers of hypoxia and immune checkpoints and found that some hypoxia marker(SLC16A1, CA9, SLC2A1, VEGFA, FOSL1, HILPDA) were up-regulated and key immune checkpoints(CTLA4, ICOS, IDO1, TNFRSF9, PD-L1, PD-1, CD86) were significantly down-regulated in m6A cluster C(**Figure 4H**).

In addition, we analyze the differences of clinical features and the enrichment of HALLMARK gene sets among three m6A clusters. The results showed that m6A cluster C was related to early initial diagnosis age, later tumor TNM stage, higher rate of progression, angiogenesis, hypoxia, P53 and PI3K pathways, whereas m6A cluster A samples showed less malignant activity(**Supplementary Figures 5B, C**).

### Distinct TNBC m6A-hypoxia phenotypes identified with differentially expressed genes

**Figure 5:**
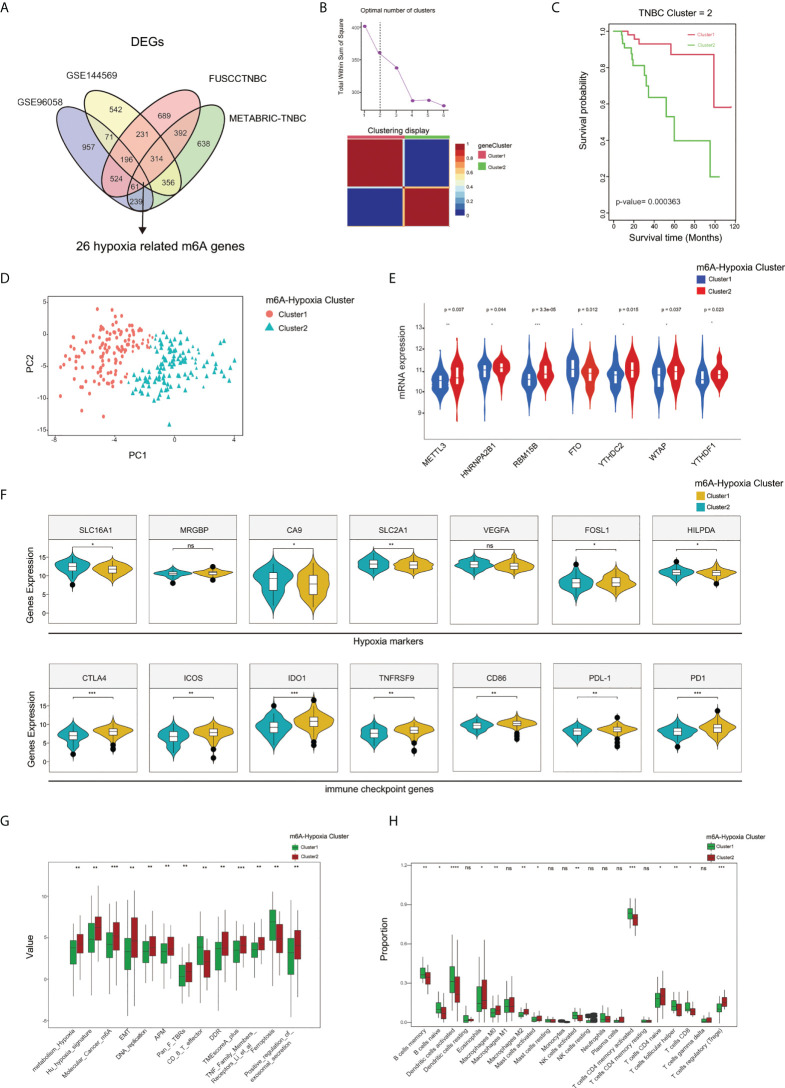
Characterization of 2 m6A-hypoxia clusters based on m6A-hypoxia related genes. **(A)** The Venn diagram indicating 26 m6A-hypoxia related genes identified in four TNBC cohorts. **(B)** Calculation number of optimal cluster and characterization of gene clusters performed by NMF method. **(C)** The overall survival analysis of m6A-hypoxia clusters. **(D)** Two gene clusters by Principal component analysis. **(E)** Comparing the expression of m6A regulators between m6A-hypoxia cluster1 and cluster2. **(F)** Comparison of hypoxia markers and immune checkpoint genes expression among 2 clusters. **(G)** Comparison of the TME and immune related signatures of 2 clusters. **(H)** Comparison of the immune infiltrated cells among different gene clusters. (*P < 0.05,**P < 0.01, ***P < 0.001, and ****P < 0.0001). ns, not significant.

We obtained hypoxia-related genes of TNBC cells cultured with 1% or 20% O_2_ from GSE144569 and intersected them with the DEGs specifically expressed among m6A clusters A, B, and C in the GEO-TNBC, METABRIC-TNBC, and FUSCCTNBC datasets, and finally get 26 m6A-hypoxia related genes (**Figure 5A**). Based on the 26 m6A-hypoxia genes, the NMF algorithm characterized TNBC samples into two m6A-hypoixa clusters, and K-M analysis showed worse overall survival in m6A-hypoxia cluster 2 compared with m6A-hypoxia cluster 1 (**Figures 5B, C**). The results of the PCA analysis were consistent with those of the above analysis and also showed clear separation of the two clusters (**Figure 5D**). We compared m6A regulators expression between the two m6A-hypoxia clusters, and six of which were differentially expressed between cluster 1 and 2(METTL3, HNRNPA2B1, RBM15B, FTO, YTHDC2, WTAP, YTHDF1), indicating that the m6-hypoxia clusters possessed distinct m6A regulation patterns(**Figure 5E**). In addition, we identified higher expression of hypoxia markers in cluster 2, whereas there was higher expression of immune checkpoint genes in m6A-hypoxia cluster1(**Figure 5F**). The analysis of TME signatures suggested that cluster 2 showed significantly higher enrichment of hypoxia, molecular_cancer_m6A, DNA replication, DDR, TMEscoreA-plus signatures, and less enrichment of CD8_T_effector and ferroptosis signatures(**Figure 5G**). Notably, while analyzing immune cell infiltration by CIBERSORT, m6A-hypoxia cluster 1 showed significantly higher proportions of memory B cells, naive B cells, activated DCs, activated NK cells, neutrophils, activated CD4 naive T cells, follicular helper T cells, and CD8 T cells than cluster 2, possibly indicating an enhanced immune response phenotype (**Figure 5H**).

### Development and prognosis value of m6A-hypoxia prognostic signature (MHPS)

**Figure 6:**
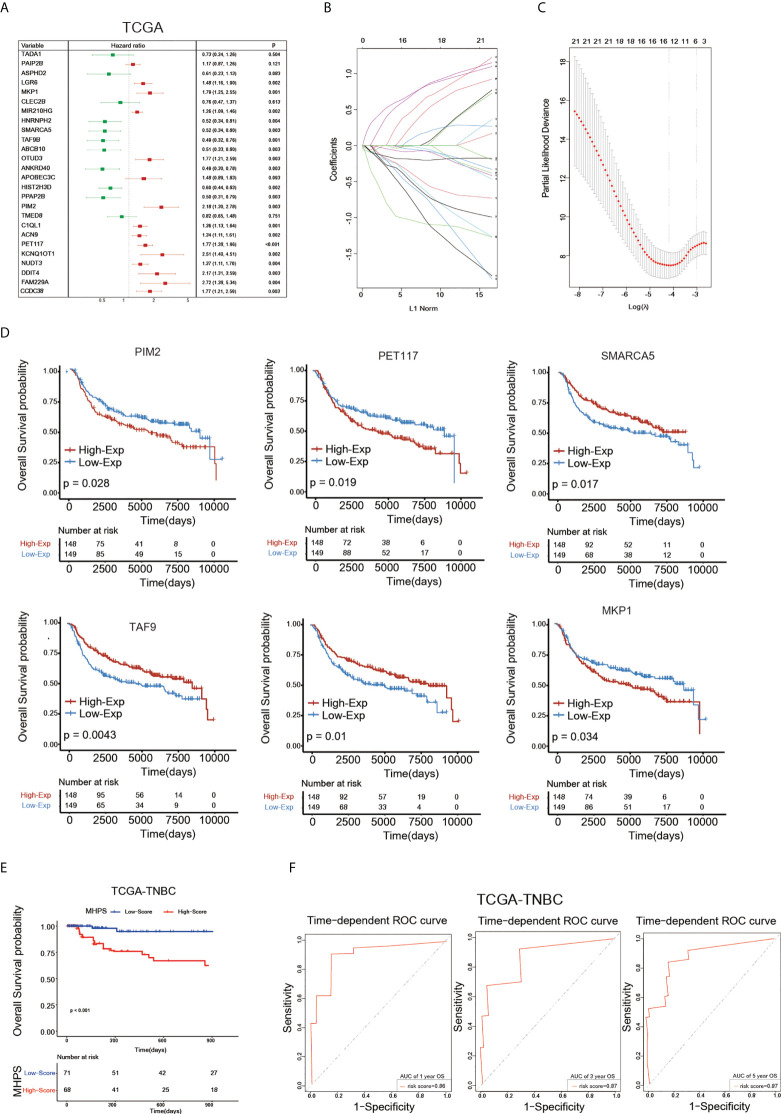
Construction of m6A-hypoxia signature(MHPS). **(A)**The Forest plot of Univariate Cox regression analysis of 26 m6A-hypoxia genes. **(B, C)** LASSO-Cox regression analysis and partial likelihood deviance of prognostic m6A-hypoxia genes. **(D)** The K-M analysis of 6 candidates gene consisting of signature. **(E)** The K-M analysis of overall survival (OS) between the high- and low-risk groups in TCGA-TNBC. **(F)** ROC curve analysis of MHPS in TNBC cohorts.

Having characterized the m6A modification pattern and TME profile in two m6A-hypoxia clusters based on 26 m6A-hypoxia genes in TNBC, we then analyzed the prognostic value of these genes. The hazard ratio of each m6A-hypoxia gene for overall survival were showed by forest plot, most of which present significant prognostic values(**Figure 6A**). Then, LASSO-Cox regression screened six hub genes to construct a molecular signature to predict the prognosis of TNBC patients (**Figure 6B**). Furthermore, K-M curves validated the prognostic values of six hub genes based on METABRIC-TNBC samples, PIM2, PET117, MKP1 were risk factors, while SMARCA5, TAF9 and ABCB10 were protective factors for TNBC prognosis(**Figure 6D**). The risk score of each TNBC sample was calculated by the MHPS model, and divided TNBC samples into high- and low-risk groups. Analysis of overall survival showed that the high-risk group had worse prognosis than the low-risk group in the TCGA-TNBC cohorts, the ROC curve showed the prognostic predictive ability for 1-, 3-, 5- years overall survival, the AUC was 0.86, 0.87 and 0.87, respectively(**Figures 6E, F**).

### Functional annotation of the MHPS TNBC subgroups

**Figure 7:**
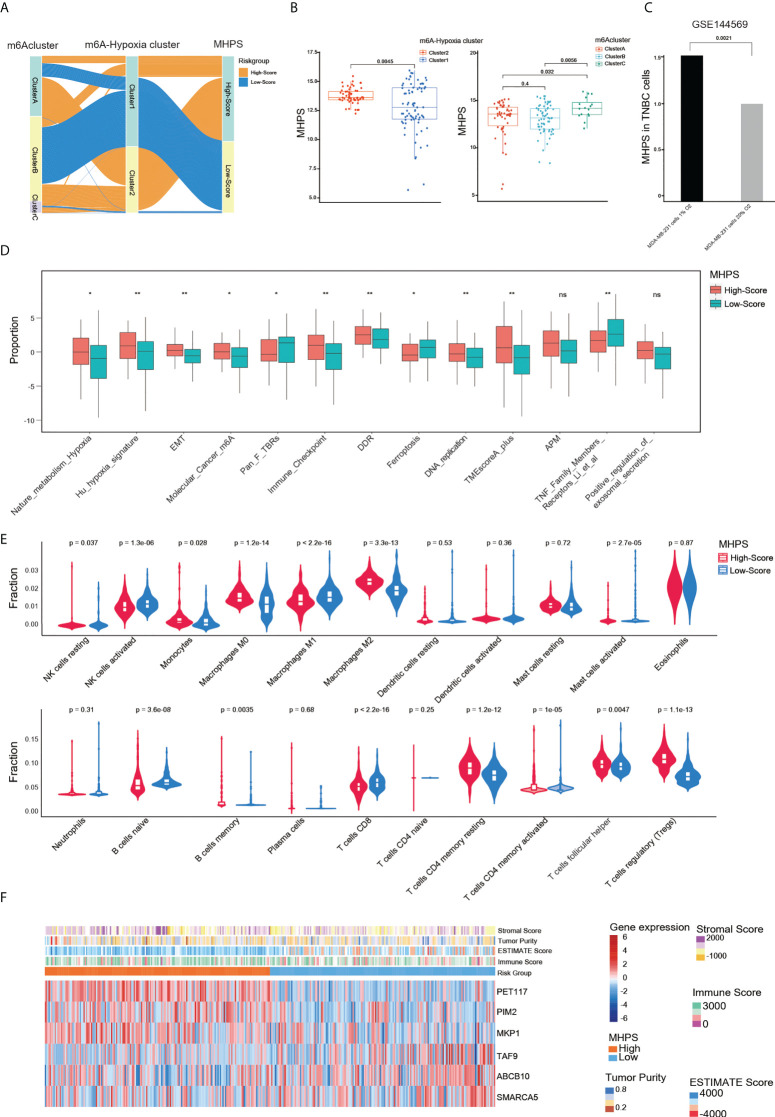
Evaluation of the clinical features of MHPS in TNBC. **(A)** Alluvial diagram of subgroup distributions in groups with different MHPS score, m6A clusters and m6A-hypoxia cluster. **(B)** Comparison of MHPS scores in 2 m6A-hypoxia and 3 m6A clusters. **(C)** Comparison of MHPS scores in MDA-MB-231 cell lines cultured under normoxia and hypoxia. **(D)** TME and hypoxia signatures score in high and low MHPS group. **(E)** Immune cell infiltration in high and low MHPS group. **(F)** Heatmap of hub gene expression profile and immune score, ESTIMATE score, stromal score and tumor purity distribution in high and low MHPS group. (*P< 0.05 and **P< 0.01). ns, not significant.

We characterized diverse subtypes of TNBC patients based on different gene expression profiles and constructed an alluvial diagram to show the associations among m6A clusters, m6A-hypoxia gene clusters, and the MHPS risk groups(**Figure 7A**). Consistent with the alluvial diagram, a box plot showed higher risk scores in m6A-hypoxia cluster 1 and m6A cluster C (**Figure 7B**). In addition, in GSE144569, the signature risk score was almost 0.5 times higher in TNBC cells cultured with hypoxia(1% O_2_) than in cells cultured with normoxia(20% O_2_), indicating that the MHPS could predict the hypoxia status of the TME (**Figure 7C**). Thirteen signatures related to tumor biological characteristics showed significantly different enrichment in the high- and low-risk groups(p<0.001). The results showed that the high-risk group had higher enrichment for hypoxia, EMT, Cancer_m6A_modification, DDR, DNA replication, antigen processing and presenting machinery (APM) signatures, whereas the low-risk group was mainly enriched for aspects of the immune system and tumor cell growth suppression, such as PAN_F_TBRs, immune checkpoints, TNF family members receptors and ferroptosis(**Figure 7D**). We also found that Tregs, monocytes, resting DCs, resting mast cells, and M2 macrophages have higher infiltrating proportions in the high-risk group, while less NK cells activated, M1 macrophages, B cells naive, CD4 and CD8 T cells infiltrated in high-risk group(**Figure 7E**). The expression profile of hub genes in TCGA-TNBC samples were visualized by heatmap. In addition, the immune score were higher in low-risk group, while tumor purity, ESTIMATE score and stromal score were higher in high-risk group(**Figure 7F**). These results suggested that MHPS-divided TNBC subgroups have significant TME and immune infiltration profiles, the high risk group present a hypoxia-related phenotype and worse anti-tumor immune response.

### Prediction of immunotherapy response with MHPS

**Figure 8:**
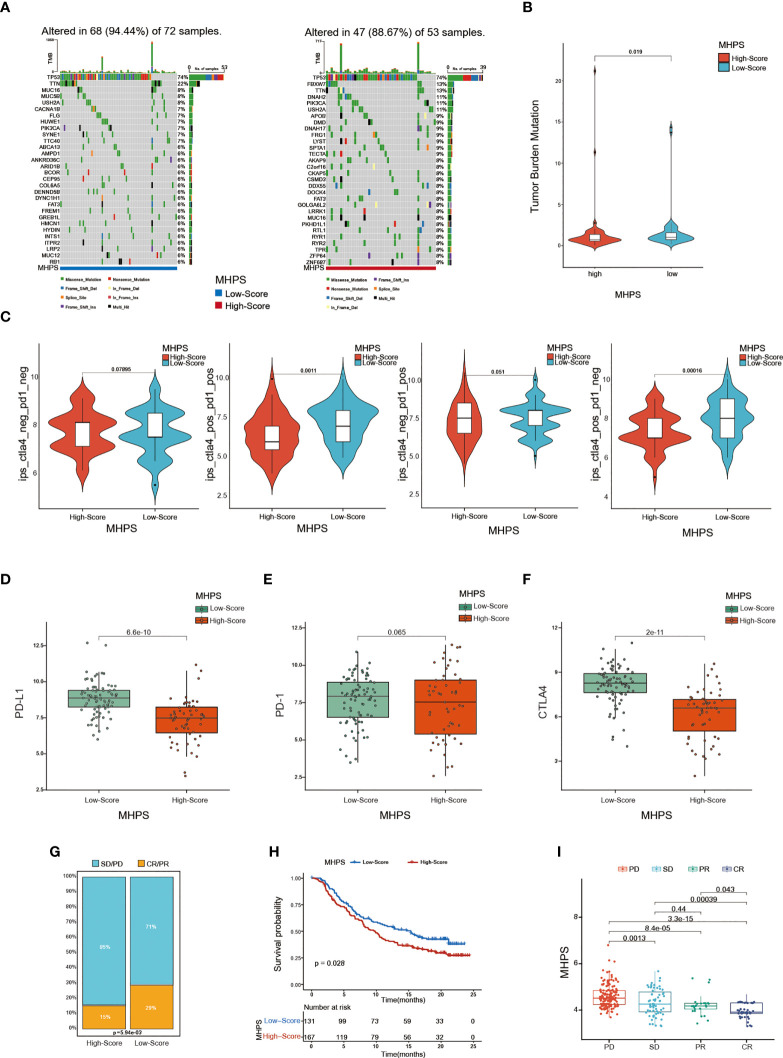
Evaluation of the sensitivity to immunotherapy with MHPS **(A)** Oncoplot of gene mutations in the low- and high-risk groups. **(B)** The tumor burden of low- and high-risk groups. **(C)** Comparison of immunophenoscore (IPS) between low and high MHPS risk groups. **(D–F)** Expression of PD-1, PD-L1, CTLA-4 in low- and high-risk groups **(G)**Proportion of immunotherapy responsive and ineffective patients in low- and high-risk groups. SD, stable disease; PD, progressive disease; CR, complete response; PR, partial response. **(H)** Different overall survival of patients with high or low MHPS score. **(I)** Comparison of risk score of patients with different immunotherapy responses.

In addition, we analyzed the somatic mutations in the high- and low-risk group, and observed that TP53 was the most frequently mutated gene in both high and low-risk group, but a higher mutation frequency of FBXW7, DNAH2, PIK3CA, USH2A genes in high-risk group(**Figure 8A**). The tumor mutational burden was higher in the low-risk group, which might have a better response to ICIs therapies(p<0.05) (**Figure 8B**). Accumulating evidence indicates that ICI therapy is an effective treatment for tumors. For instance, the Food and Drug Administration has approved pembrolizumab for PD-1 inhibition, and CTLA-4 inhibitors are now in clinical trials for TNBC (35). Therefore, we assessed the immunophenotype score(IPS) to determine the correlation between risk score and response to ICIs in TNBC patients. We found that the IPS was higher in the low-risk group than in the high-risk group for patients treated with CTLA-4 or PD-1 inhibitors or both (**Figure 8C**). Moreover, the low-risk group showed higher expression of important immune checkpoint biomarkers (PD-1, PD-L1, and CTLA-4) compared with the high-risk group (**Figures 8D**–**F**). Otherwise, to evaluate the ability of MHPS in predicting response of immunotherapy, we analyzed the patients from IMvigor210 cohort of who received the anti-PD-L1 antibody Atezolizumab treatment (36) and found that the less number of patients responded to anti-PDL1 treatment in high-risk group and the patients have a significant survival disadvantage in high-risk group(**Figures 8G, H**). The patients of progressive disease(PD) obtain higher risk scores than partial response(PR) and complete response(CR) patients (**Figure 8I**). Taken together, the above results indicate that our m6A-hypoxia score is associated with response to immunotherapy.

### Construction and validation of nomogram base on MHPS and detection of hub genes expression

**Figure 9:**
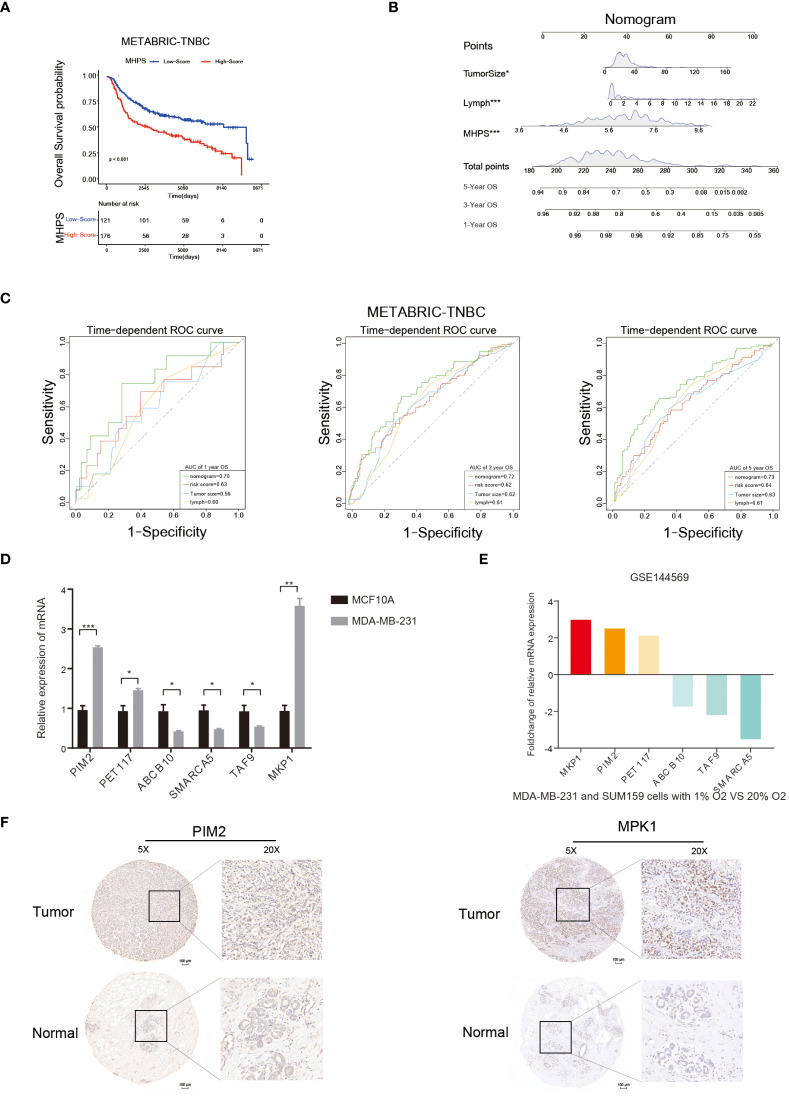
Nomogram and detection of MHPS gene expression. **(A)** Kaplan-Meier analysis of patients with high or low MHPS risk socre in METABRIC-TNBC cohort. **(B)** Construction of nomogram scoring system to predict patient survival at 1-, 3- and 5- years. Each clinical factor in the nomogram system corresponds to a score, and all scores are summed to obtain a total point, which can predict the survival rate of patients at 1-, 3- and 5- years. **(C)** Time-dependent ROC for the nomogram, MHPS, tumor size, lymph node in the METABRIC cohort (for predicting 1, 3, and 5-years OS). **(D)** Comparison of mRNA expression of hub genes in normal breast and TNBC cell lines. **(E)** Different expression of hub genes in normoxia and hypoxia cultured TNBC cells based on GSE144569 dataset. **(F)** IHC staining to detect protein expression of PIM2 and MKP1 in normal and tumor tissues. Scale bar:100μm.(*P< 0.05,**P< 0.01, and ***P< 0.001). ns, not significant.

Kaplan-Meier curves and log-rank test showed that patients in the high-MHPS group had significantly worse prognosis than low-MHPS group in METABRIC-TNBC cohort(**Figure 9A**). To affiliated the clinical application of MHPS, we combined the m6A-hypoxia signature score with clinical prognostic factors (tumor size, lymph node) in METABRIC-TNBC cohort and established a nomogram to estimate 1-, 3-, and 5-year overall survival of TNBC patients(**Figure 9B**). Time-dependent ROC curves were used to evaluate the predictive efficacy of the prognostic factors. The results showed that nomogram was better than other factors (including risk score, lymph node and tumor size).The AUCs of 1-, 3-, and 5-years reached 0.70, 0.72, and 0.73 (1-, 3- and 5-years) in METABRIC cohort (**Figures 9C**). These results indicated that the nomogram based on MHPS, tumor size and lymph nodes has a strong and stable ability to predict the OS of TNBC patients.

Moreover, we performed *in vivo* and *in vitro* assays to detect the expression of hub genes in MHPS. The mRNA expression level of six hub genes in TNBC cell lines(MDA-MB-231) and normal breast cell lines(MCF10A) were measured by qRT-PCR. The results showed that PIM2, PET117, MKP1 were significantly higher expressed in MDA-MB-231 than MCF10A, while ABCB10, SMARCA5 and TAF9 were lower expressed in TNBC cell lines(**Figure 9D**). Distinct mRNA expression of hub genes were observed in TNBC cell lines explosed to normoxia or hypoxia(**Figure 9E**). Similarly, the protein expression level of PIM2 and MKP1 were higher in TNBC tissues than in adjacent normal tissues by immunohistochemical staining(**Figure 9F**).

## Discussion

m6A RNA methylation is a form of epigenetic regulation that has been shown to affect various aspects of RNA metabolism, including pre-mRNA splicing, 3’-end processing, nuclear export, translation regulation, mRNA decay, and noncoding RNA processing, and can participate in tumor initiation and development (37). Further exploration of the mechanisms underlying m6A modification in tumors showed that it was mediated by methyltransferases (writers), demethylases (erasers), and m6A-binding proteins (readers) (38). The writers (METTL3, METL14, METL16, WTAP, KIAA1429, ZC3H13, RBM15, and RBM15B) catalyze m6A modification of adenosine on mRNA, the erasers (FTO and ALKBH5) remove the adenosine from mRNA, and the readers (YTHDC1, YTHDC2, YTHDF1, YTHDF2, YTHDF3, HNRNPC, HNRNPA2B1, IGFBP1, IGFBP2, IGFBP3, and RBMX) recognize m6A modification sites and produce corresponding biological effects (39). Jaclyn et al. demonstrated that depletion of YTHDF2 mRNA degradation triggered apoptosis in TNBC cells and tumors through the MAPK pathway (40). Shi et al. also revealed that METTL3 influenced TNBC metastasis through m6A modification of COL3A1 (41). In our study, through analysis of 255 signatures in TNBC, we found that an m6A signature and other signatures scored higher in TNBC than in normal tissue and found that m6A had positive relationship with hypoxia and immune infiltration signatures. Therefore, based on several studies on m6A modification, we attempted to identify clinical features and TME characteristics associated with different m6A modification patterns for the exploration of immunotherapy strategies in TNBC.

For the analysis of gene expression, we collected 46 m6A genes including 21 m6A regulators and 25 m6A-related genes and found that most of them were differently expressed between TNBC and normal tissue; moreover, this differential expression pattern could be used to characterize three m6A clusters by NMF. To explore different m6A modification patterns in TNBC, firstly, we identified significant differences in prognosis among the three clusters, with worse overall survival in m6A cluster C than in m6A clusters A and B. Then, by ssGSEA of 255 signatures in the three m6A clusters, we found m6A cluster C mainly related to metabolism and hypoxia signatures. The activities of metabolism include the methionine cycle, which generates S-adenosylmethionine and participates in m6A modification (42), glycerolipid metabolism, which is critical for homeostasis of cellular lipid stores and membranes (43); and glycolysis, the progress of which is regulated by HIFs and leads to tumor hypoxia TME (44). Moreover, we identified immune infiltration in m6A cluster B with activation of adaptive immunity is considered to indicate an inflammatory immune phenotype. By contrast, the immune cell infiltration profile found in m6A cluster C indicated an immune-excluded phenotype of TNBC. However, we were unable to identify any distinguishing characteristics of m6A cluster A in our study.

The TME has an important role in tumor progression. Wei et al. (45) revealed that metabolism reprogramming was related to the hypoxic microenvironment and affected immune cell infiltration. Combined with the results above, we explored the characteristics of both m6A modification and hypoxia status in TNBC. Therefore, based on a comparison of gene expression in three m6A modification clusters, we identified 26 genes related to both m6A and hypoxia regulation and used them to successfully characterize two gene clusters. Analysis of prognosis between the two groups indicated significantly worse prognosis in gene cluster 2. Considering the tumor heterogeneity of the two clusters, we identified six prognostic genes (PIM2, PET117, SMARCA5, TAF9, ABCB10, MKP1) among the m6A-hypoxia genes and developed a scoring system to evaluate m6A modification patterns and hypoxia status. Zhou et al. clarified that MKP-1 increased levels of phosphorylated p38 and JNK and inhibited cell death in breast cancer (46). Seta et al. reported that MKP-1 expression was upregulated by hypoxia (47). Thus, the relationship between MKP-1 and m6A modification in TNBC would be worth exploration in the future. PIM2 is a oncogene that have been validated in breast, liver cancer and chronic lymphocytic leukemia (48, 49). Tingting Yang et al. demonstrated that PIM2 mediated phosphorylation of HSF1 at Thr120 to regulate HSF1 protein stability, and induce PD-L1 expression in breast cancer, which suggested that it might affect immune therapy response (50). Researches about PET117 indicated that it is necessary for Cox15 oligomerization and function in mitochondrial respiratory (51), which means it might related to cell energy metabolism. There was a bioinformatic analyse identified PET117 as hub gene related to TME and prognosis of hepatocellular Carcinoma (52). The TAF9(TATA-binding protein) was also identified as a prognostic gene in a DNA-repair-related gene model for esophageal cance (53), our results showed that MHPS was also associated DDR, which suggested that TAF9 might involved in tumor processes, and discovered as a potential target in TNBC. SMARCA5 is a chromatin-remodeling enzyme which has been reported related to Keap1-Nrf2 signaling. But its role in cancer is still undiscovered, which needs further exploration (54). ABCB10(ABC Transporter 10 Protein) is a member of the MDR/TAP subfamily, which are involved in multidrug resistance but the function of ABCB10 require further identification (55). Thus, the MHPS included several oncogenic genes related to TME, DDR and immune therapy response validated in different cancer types, and also provide some novel genes that worth further exploration of their function in TNBC and other cancers.

Further more, by CIBERSORT analysis, we identified a significant relationship between immune cell infiltration and the MHPS. Higher proportions of Tregs, monocytes, resting DCs, resting mast cells, and M2 macrophages were found in the high-risk group compared with the low-risk group. The function of classically activated M1 macrophages is inhibition of tumor development, whereas alternatively activated M2 macrophages may promote tumor proliferation and invasion (56). The PD-1/PD-L pathway, which inhibits T cell activation and proliferation, and CTLA-4, which can induce cell-cycle arrest and apoptosis-activated T cells, have been widely investigated as targets for immunotherapy (57). According to the differential expression of PD-L1, PD-1, and CTLA-4 between the high- and low-risk groups, we predicted that immune treatment would tend to be more beneficial in the low-risk group. The result was proved in IMvigor210 cohort. Thus, combining m6A modification patterns with expression of hypoxia-related genes may represent a novel and efficient classifier of characteristics of the immune microenvironment for prediction of immune response and overall survival of TNBC patients (58, 59).

In conclusion, this exploration of m6A modifications and hypoxia-related genes demonstrated different characteristics of the TNBC immune microenvironment. On this basis, a gene signature was developed for accurate prediction of immune therapy response and prognosis of TNBC patients. These findings may help to advance our understanding of the association between m6A modification and the TME and provide new approaches to individual therapy for TNBC patients.

## Data availability statement

The original contributions presented in the study are included in the article/**Supplementary Material**. Further inquiries can be directed to the corresponding author.

## Ethics statement

The studies involving human participants were reviewed and approved by Medical Ethics Committee of Peking University Cancer Hospital (No. 2020KT75). The patients/participants provided their written informed consent to participate in this study.

## Author contributions

All authors contributed to the study’s conception and design. Material preparation, data collection, and analysis were performed by XS and JZ. The first draft of the manuscript was written by XS and all authors commented on previous versions of the manuscript. All authors read and approved the final manuscript. All authors contributed to the article and approved the submitted version.

## Funding

This research was supported by Science and Technology Department of Sichuan Province. Grant number: 2019YFS0379.

## Conflict of interest

The authors declare that the research was conducted in the absence of any commercial or financial relationships that could be construed as a potential conflict of interest.

## Publisher’s note

All claims expressed in this article are solely those of the authors and do not necessarily represent those of their affiliated organizations, or those of the publisher, the editors and the reviewers. Any product that may be evaluated in this article, or claim that may be made by its manufacturer, is not guaranteed or endorsed by the publisher.
