# Corrigendum: Identification of m6A modification patterns and development of m6A–hypoxia prognostic signature to characterize tumor microenvironment in triple-negative breast cancer

**DOI:** 10.3389/fimmu.2024.1441843

**Published:** 2024-07-05

**Authors:** Xi Shen, Jianxin Zhong, Jinlan He, Jiaqi Han, Nianyong Chen

**Affiliations:** ^1^ Department of Head and Neck Oncology and Department of Radiation Oncology, Cancer Center, West China Hospital, Sichuan University, Chengdu, China; ^2^ Department of Breast Oncology, Key Laboratory of Carcinogenesis and Translational Research (Ministry of Education), Peking University Cancer Hospital & Institute, Beijing, China

**Keywords:** Triple-negative breast cancer, m6A RNA methylation, m6A-hypoxia signature, tumor microenvironment, immune cell infiltration

In the published article, there was an error in **Figure 9F** as published. After checking the original paper, the images of **Figure 9F** were mistakenly included. The corrected **Figure 9** and its caption **Figure 9** Nomogram and detection of MHPS gene expression. appear below.

**Figure 9:**
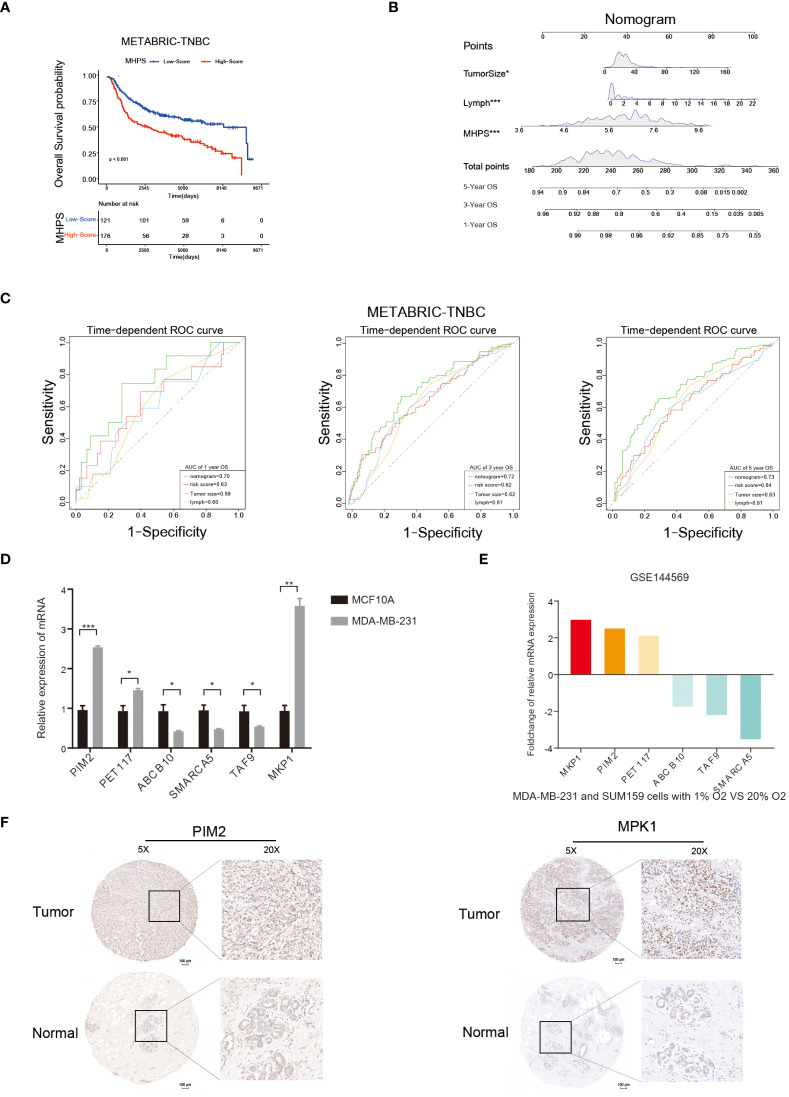
Nomogram and detection of MHPS gene expression. **(A)** Kaplan-Meier analysis of patients with high or low MHPS risk socre in METABRIC-TNBC cohort. **(B)** Construction of nomogram scoring system to predict patient survival at 1-, 3- and 5- years. Each clinical factor in the nomogram system corresponds to a score, and all scores are summed to obtain a total point, which can predict the survival rate of patients at 1-, 3- and 5- years. **(C)** Time-dependent ROC for the nomogram, MHPS, tumor size, lymph node in the METABRIC cohort (for predicting 1, 3, and 5-years OS). **(D)** Comparison of mRNA expression of hub genes in normal breast and TNBC cell lines. **(E)** Different expression of hub genes in normoxia and hypoxia cultured TNBC cells based on GSE144569 dataset. **(F)** IHC staining to detect protein expression of PIM2 and MKP1 in normal and tumor tissues. Scale bar:100μm.(*P< 0.05,**P< 0.01, and ***P< 0.001). ns, not significant.

The authors apologize for this error and state that this does not change the scientific conclusions of the article in any way. The original article has been updated.

