# Supplementary figures and images for: Identification of m6A modification patterns and development of m6A–hypoxia prognostic signature to characterize tumor microenvironment in triple-negative breast cancer
Source: Front Immunol. 2022 Aug 29;13:978092. doi: 10.3389/fimmu.2022.978092 (PMC9465332; doi:10.3389/fimmu.2022.978092)

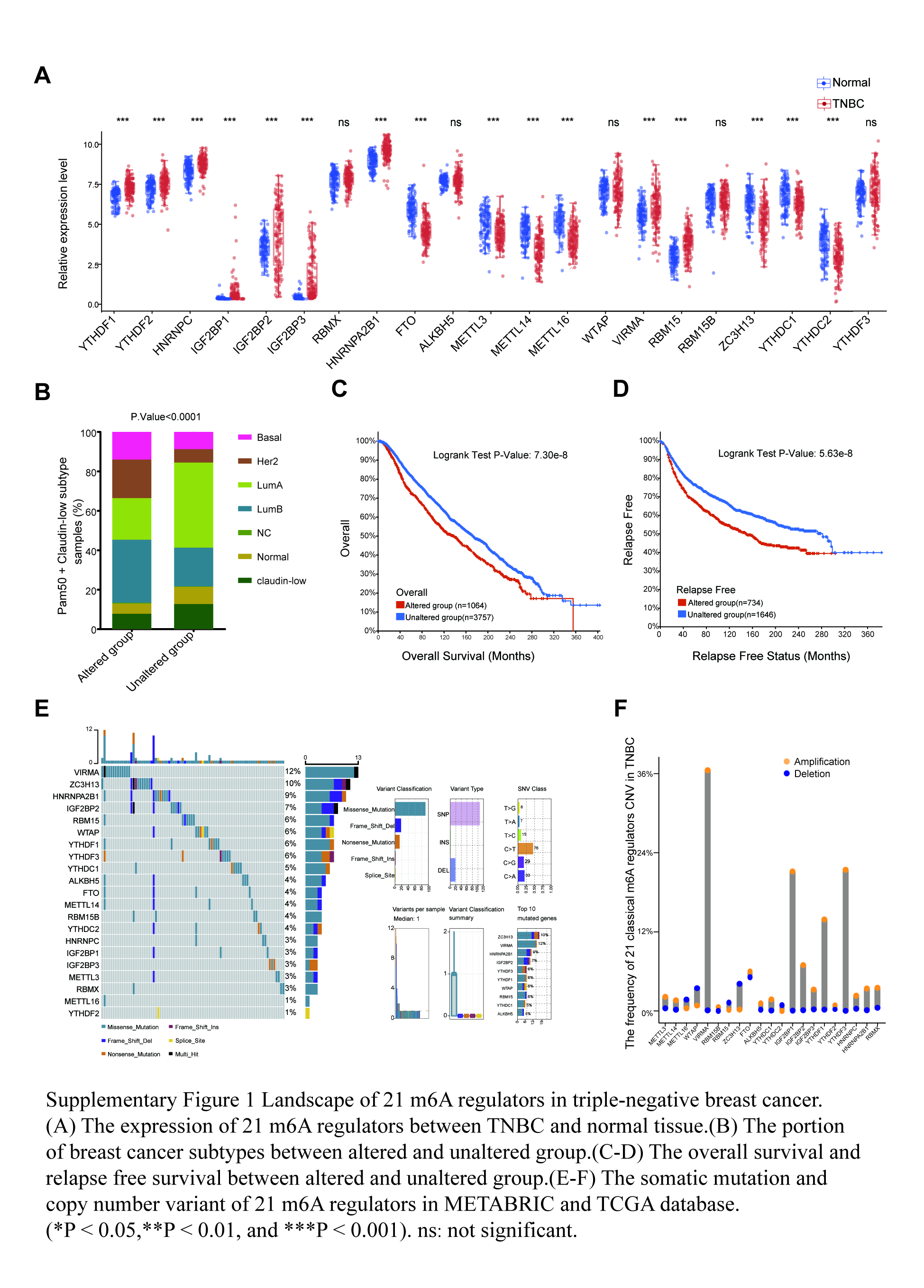

Supplement: Supplementary file 1 [file Image_1.tif]

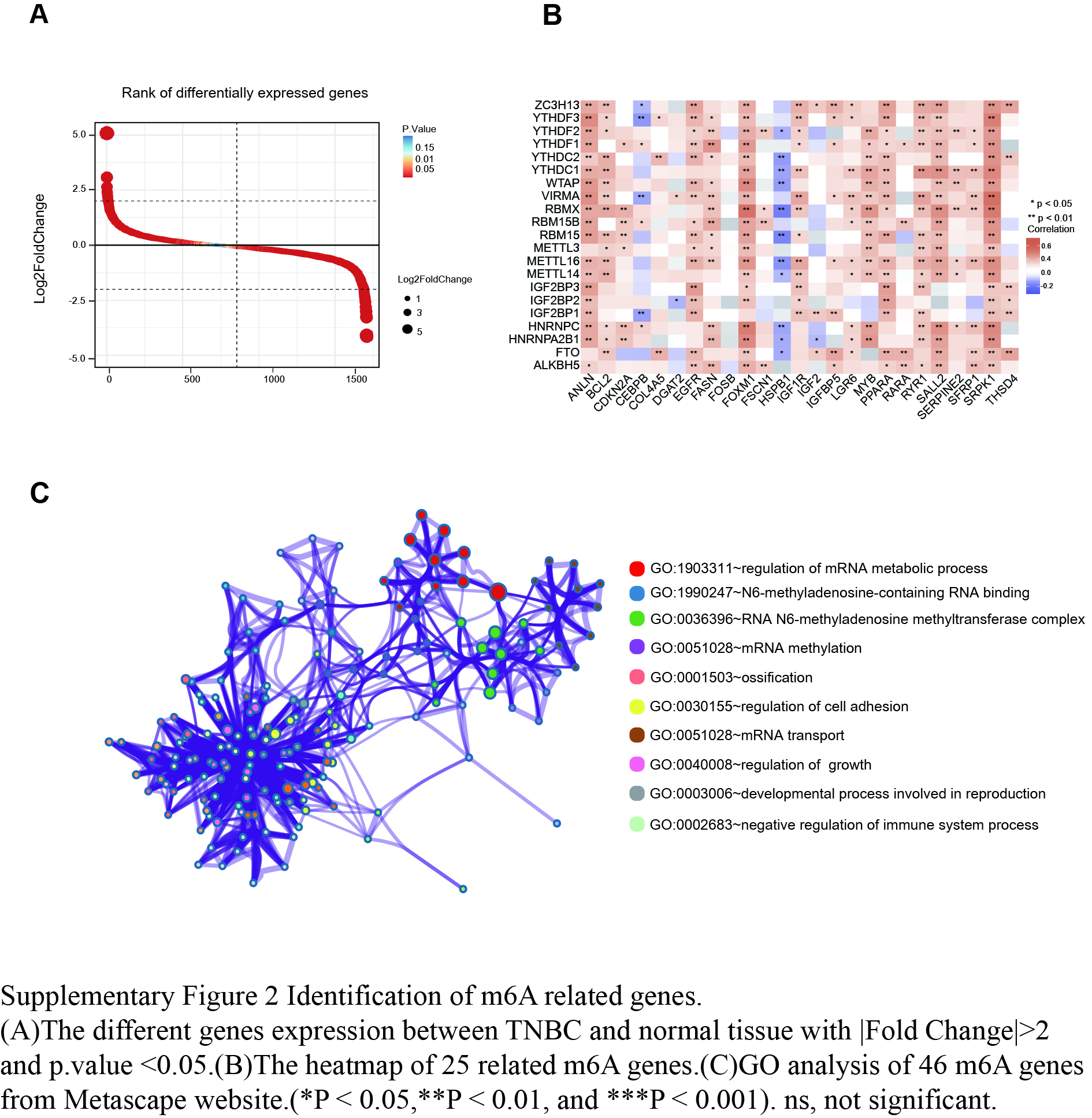

Supplement: Supplementary file 2 [file Image_2.tif]

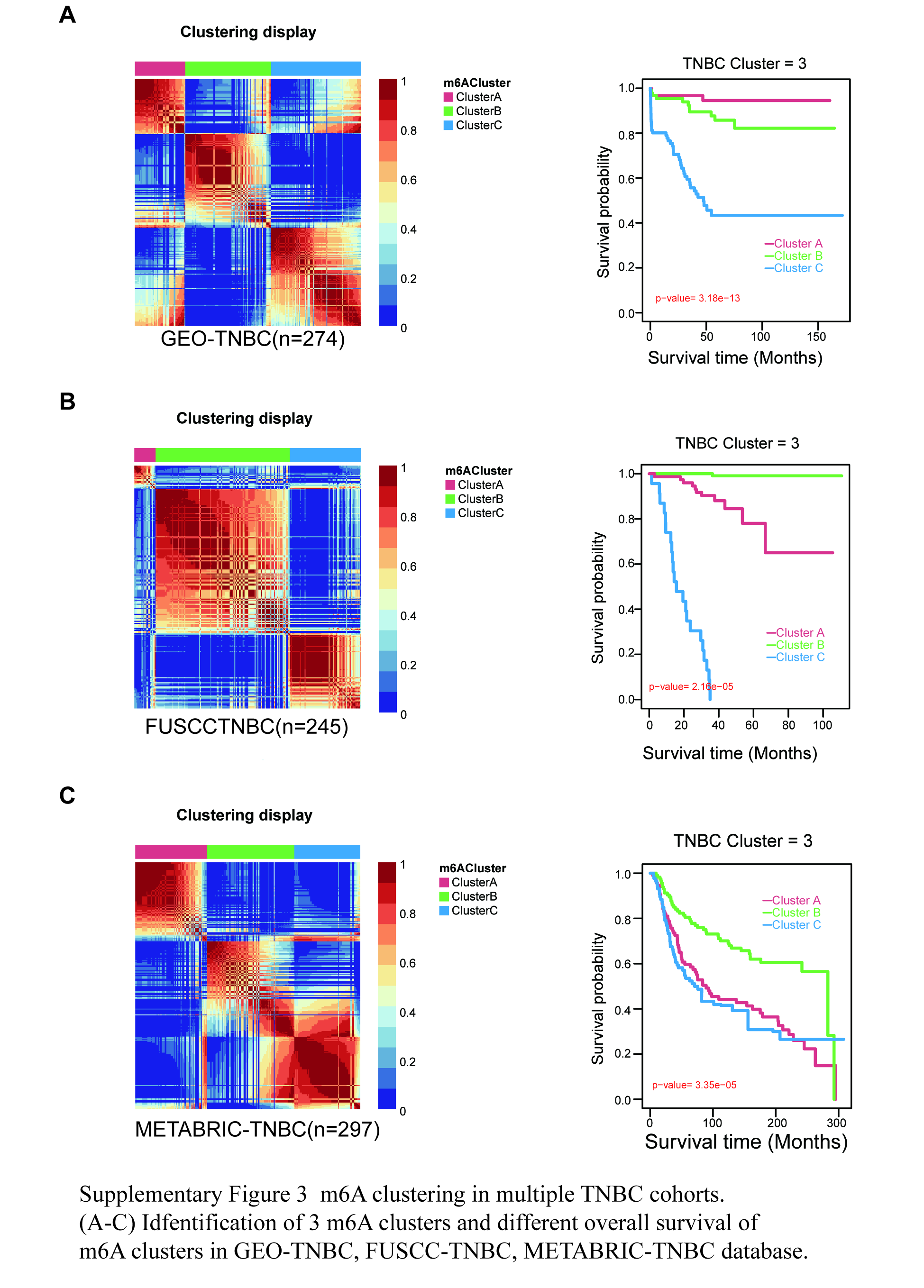

Supplement: Supplementary file 3 [file Image_3.tif]

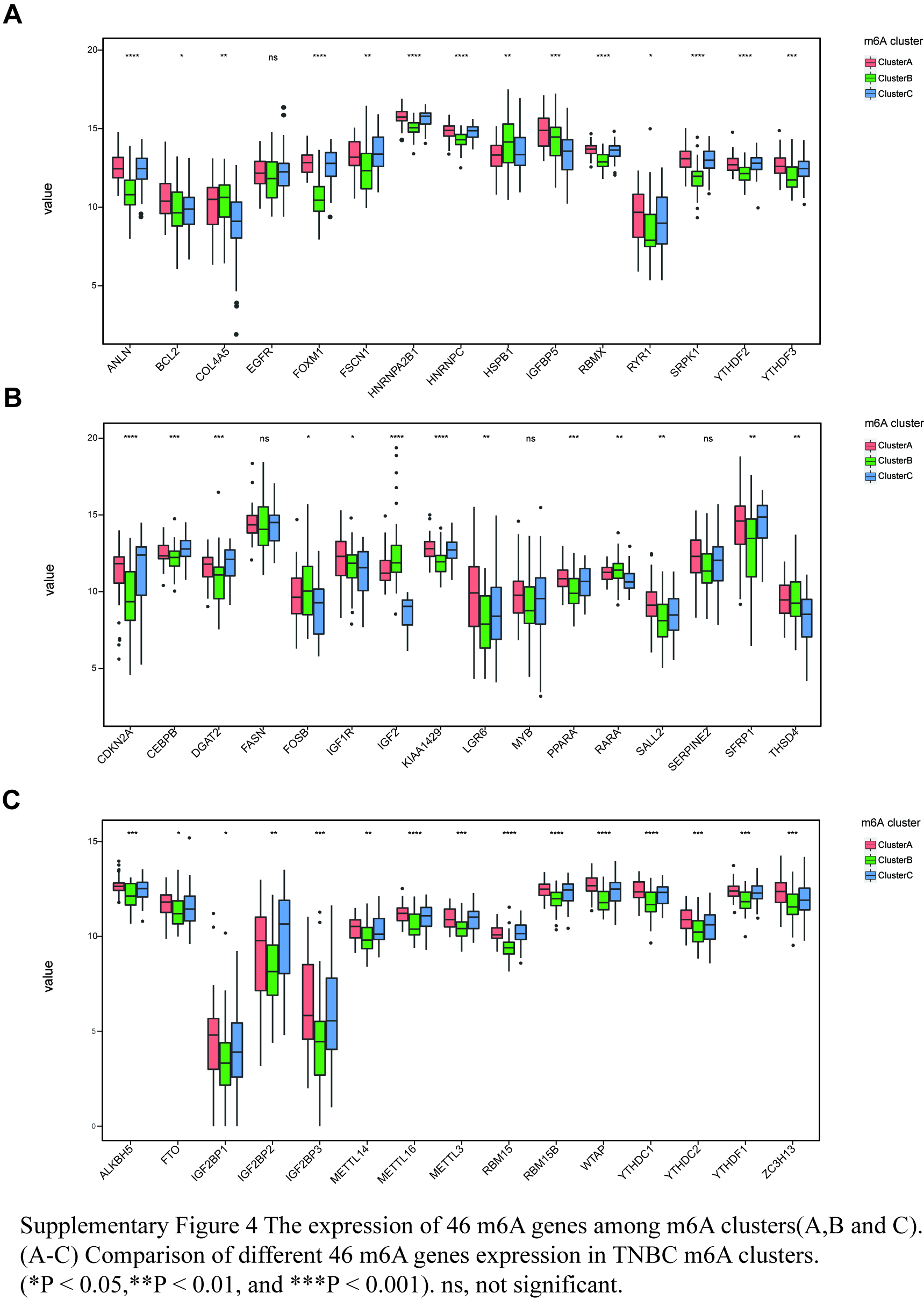

Supplement: Supplementary file 4 [file Image_4.tif]

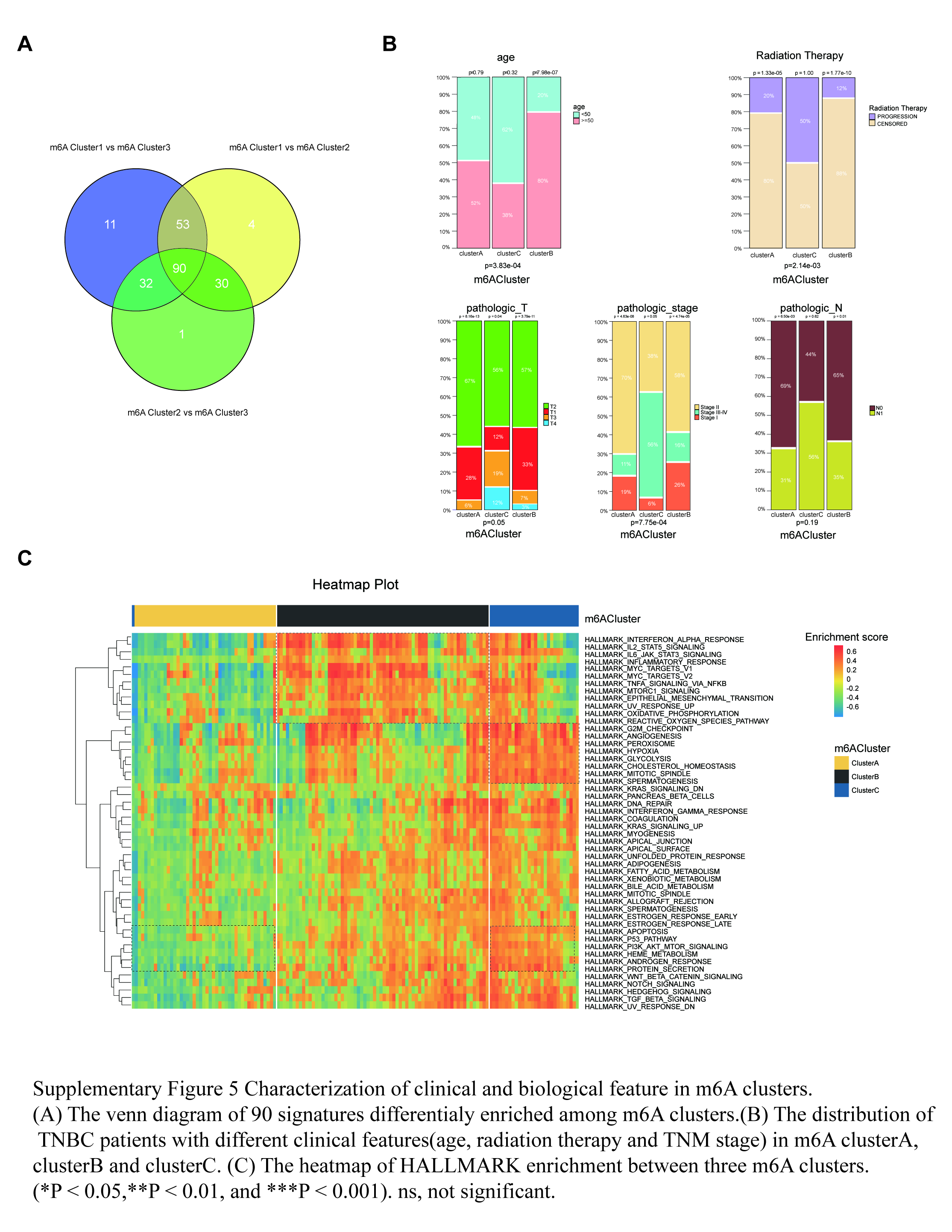

Supplement: Supplementary file 5 [file Image_5.tif]
